# Ca^2+^-Dependent Cl^−^ Channels in Vascular Tone Regulation during Aging

**DOI:** 10.3390/ijms25105093

**Published:** 2024-05-07

**Authors:** Miriam Petrova, Monika Lassanova, Jana Tisonova, Silvia Liskova

**Affiliations:** 1 Faculty of Medicine, Institute of Pharmacology and Clinical Pharmacology, Comenius University, Sasinkova 4, 811 08 Bratislava, Slovakia; miriam.petrova@fmed.uniba.sk (M.P.); monika.lassanova@fmed.uniba.sk (M.L.); jana.tisonova@fmed.uniba.sk (J.T.); 2 Centre of Experimental Medicine, v.v.i., Institute of Normal and Pathological Physiology, Slovak Academy of Sciences, Sienkiewiczova 1, 813 71 Bratislava, Slovakia

**Keywords:** Ca^2+^-dependent Cl^−^ channels, aging, vascular tone regulation, sympathetic nerve activity, phospholipid scramblase, TMEM16 protein family

## Abstract

Identifying alterations caused by aging could be an important tool for improving the diagnosis of cardiovascular diseases. Changes in vascular tone regulation involve various mechanisms, like NO synthase activity, activity of the sympathetic nervous system, production of prostaglandin, endothelium-dependent relaxing, and contracting factors, etc. Surprisingly, Ca^2+^-dependent Cl^−^ channels (CaCCs) are involved in all alterations of the vascular tone regulation mentioned above. Furthermore, we discuss these mechanisms in the context of ontogenetic development and aging. The molecular and electrophysiological mechanisms of CaCCs activation on the cell membrane of the vascular smooth muscle cells (VSMC) and endothelium are explained, as well as the age-dependent changes that imply the activation or inhibition of CaCCs. In conclusion, due to the diverse intracellular concentration of chloride in VSMC and endothelial cells, the activation of CaCCs depends, in part, on intracellular Ca^2+^ concentration, and, in part, on voltage, leading to fine adjustments of vascular tone. The activation of CaCCs declines during ontogenetic development and aging. This decline in the activation of CaCCs involves a decrease in protein level, the impairment of Ca^2+^ influx, and probably other alterations in vascular tone regulation.

## 1. Introduction

Aging is the main risk factor for the development of cardiovascular diseases. Vascular aging is linked to hypertension, and at the same time, increased blood pressure accelerates vascular aging. This is why determination of the alteration caused by vascular aging is important for cardiovascular prevention [1]. Mechanisms involved in vascular aging require further investigation, but experimental studies are sparse. Experiments with aged animals require longer time and more animals per group due to the higher possibility of a lower survival rate.

In central and peripheral arteries, age-dependent changes are associated with a progressive decline in vascular responses to constrictors [2]. Several other mechanisms involved in age-dependent vascular changes have been described, such as impaired nitric oxide synthase (NOS) activity [3,4], activation of Rho kinase [2,5], alterations in sympathetic nerve activity (SNA) [6,7], adrenergic regulation [8,9], increased vascular endothelin levels [10], alteration in prostaglandins production or release [11,12], and sexual blood pressure dimorphism [13].

In the presented review, we disclose the link between various mechanisms relevant to ontogenetic development and aging in relation to Ca^2+^-dependent Cl^−^ channels (CaCCs). During the last decade of cardiovascular research, age-dependent changes have been evaluated in order to disclose mechanisms relevant in the aged population. However, there is only a little information about the involvement of aging in the regulation of vascular tone that consider the contribution of CaCCs. One reason may be that cytosolic Cl^−^ concentration varies in a range from 10 to 60 mmol/L depending on the tissue [14]. In addition to changes in cytosolic Cl^−^ concentration that affect CaCCs-dependent currents, CaCCs have been shown to have phospholipid scramblase activity [15], and the presence of CaCCs decreases in the vasculature due to sympathetic nerve development during ontogenetic development [16]. 

In 2008, it was found that at the molecular level, functional CaCCs are formed by proteins belonging to the anoctamin/transmembrane protein 16 (TMEM16) family [17,18,19]. Among the many diverse physiological functions where the TMEM16 protein family is involved include vascular tone regulation, coagulation, proliferation, apoptosis, angiogenesis, tumorigenesis, etc. 

## 2. Ca^2+^-Dependent Cl^−^ Channels of Vascular Smooth Muscle Cell during Aging

CaCCs are exceptionally well adapted to serve diverse physiological roles. The [Cl^−^]i in the vascular smooth muscle cell (VSMC) was shown to range from 30 mmol/L to 50 mmol/L [20]. More precisely, it was reported to be about 35 mmol/L [21]. The estimated equilibrium potential for Cl^−^ is between −30 mV and −20 mV in VSMC [20]. The physiological membrane potential of VSMC is within the range of −60 mV to −40 mV [22]. Thus, Cl^−^ channel activation in VSMC results in Cl^−^ efflux, leading to membrane depolarization and voltage-dependent calcium channel activation and contraction [23]. CaCCs are highly sensitive to changes in the [Ca^2+^]i, but CaCCs are also synergistically gated by Ca^2+^ and voltage [24].

**Figure 1:**
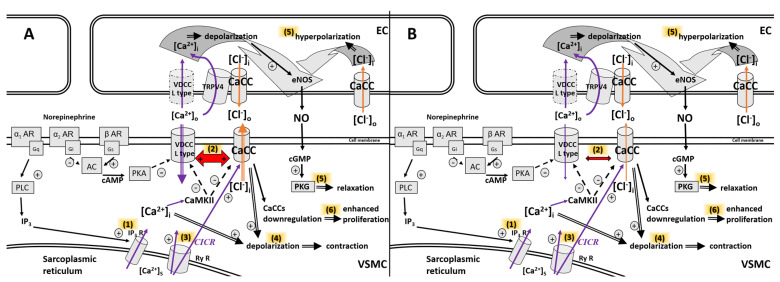
The activation of CaCCs during agonist (norepinephrine)–induced contraction in the arteries of young (**A**) and aged (**B**) animals. (**1**) The release of Ca^2+^ from sarcoplasmic reticulum through IP_3_R leads to activation of CaCCs. (**2**) The activation of CaCCs, together with the opening of L–VDCC (positive feedback, red arrow), causing fast depolarization of VSMC via the Cl^−^ efflux and Ca^2+^ influx (**A**). Age-dependent changes involve a decrease in the activity of CaCCs and L–VDCC, causing slower depolarization of VSMC (**B**). (**3**) Elevated [Ca^2+^]i activates CICR, and Ca^2+^ sparks provide additional stimulus for CaCCs activation in VSMC. (**4**) Cl^−^ efflux and Ca^2+^ influx increase depolarization and cause VSMC contraction. CaCCs and L–VDCC are inactivated through CaMKII. (**5**) Depending on the [Cl^−^]i, the activation of CaCCs on endothelium causes depolarization that increases NO release, or hyperpolarization that decreases NO release. L–VDCC are not expressed on endothelial cells (indicated by dashed lines), but the Ca^2+^ entry through L–VDCC in VSMC can pass to the endothelium through positions aligned with holes in the internal elastic lamina in amounts sufficient to activate Ca^2+^ signaling in endothelial cells [30]. TRPV4 channels are localized in nanoscale proximity of CaCCs and are activated together with CaCCs, leading to increased Ca^2+^ influx into the endothelial cell [31]. (**6**) The downregulation of CaCCs contributes to enhanced proliferation of VSMC. Solid arrows indicate stimulation, dashed arrows indicate inhibition, red arrow indicates positive feedback, purple arrows indicate Ca^2+^ currents, and orange arrows indicate Cl^−^ currents. Abbreviations: AC, adenylate cyclase; AR, adrenergic receptor; CaCCs, Ca^2+^–dependent Cl^−^ channels; cAMP, cyclic adenosine monophosphate; CIRC, Ca^2+^–induced Ca^2+^ release; CaMKII, Ca^2+^/calmodulin–dependent protein kinase II; EC, endothelial cell; eNOS, endothelial NO synthase; Gi, G protein–coupled receptor with αi subunit; Gq, G protein–coupled receptor with αq subunit; Gs, G protein–coupled receptor with αs subunit; IP_3_, inositol triphosphate; IP_3_R, IP_3_ receptor; NO, nitric oxide; L–VDCC, L–type voltage–dependent Ca^2+^ channels; PKA, protein kinase A; PKG, protein kinase G; PLC, phospholipase C; RyR, ryanodine receptor; TRPV4, transient receptor potential cation channel subfamily V member 4; VSMC, vascular smooth muscle cell.

In the vasculature, CaCCs are involved in the adjustment of vascular tone (Figure 1A). The results of various studies revealed that the contraction of VSMC involves CaCCs activation. It was shown that when TMEM16A protein was ablated on the cell membrane (through the control of the smooth myosin heavy chain promoter) and CaCCs conductance was abolished in blood vessels, the arterial blood pressure in mice decreased, and the CaCCs-dependent drop in blood pressure was about 10 mmHg [25]. CaCCs participate in norepinephrine-induced contraction of the femoral artery because the norepinephrine-induced concentration response curves were almost abolished by R(+)-IAA-94 (CaCCs inhibitor) in the femoral arteries of Wistar-Kyoto rats (WKY), as well as in the femoral arteries of spontaneously hypertensive rats (SHR) [26]. These findings are in agreement with the proposed regulation of CaCCs activation shown in Figure 1A, where the opening of CaCCs, together with opening of the L-type voltage-dependent calcium channel (L-VDCC), leads to depolarization and contraction. 

Contrary to these observations, some other studies did not find the involvement of CaCCs in the contraction of VSMC. The results of [27] showed that the stimulation of TMEM16A does not elicit augmented baseline mucin secretion, and neither does it induce bronchoconstriction nor cause pulmonary artery contraction; furthermore, the inhibition of TMEM16A did not relax human bronchial smooth muscle cells. 

Several inhibitors of CaCCs (CaCC_inh_-A01, MONNA) cause other physiological effects like preventing the increase in the [Ca^2+^]i that is created after the activation of inositol triphosphate receptors (IP_3_R) on intracellular Ca^2+^ stores [28]. Other inhibitors of CaCCs (niclosamide) lead to a decrease in the [Ca^2+^]i to the same extent as induced by inhibiting the sarcoplasmic/endoplasmic reticulum Ca^2+^-ATPase (SERCA) pump [28]. Thus, signaling pathways suggested by studies that used CaCCs inhibitors need further investigation. 

Only few studies evaluated the CaCCs during aging. The norepinephrine-induced concentration response curves of the femoral artery were almost abolished by R(+)-IAA-94 (CaCCs inhibitor) in 6-months-old WKY and SHR and 12-months-old WKY, but in 12-months-old SHR, the CaCCs inhibitor caused only a 50% reduction of norepinephrine-induced concentration response curves of the femoral artery [26]. Using 20-months-old WKY rats, the norepinephrine-induced concentration response curves of the femoral artery were not influenced by the application of CaCCs inhibitor [26], showing an age-dependent decline in CaCCs activity in the femoral artery of normotensive and hypertensive rats.

Similarly, a decline in CaCCs activity in hypertensive rats was shown in [29], where it was also shown that TMEM16A-mediated CaCCs is functioning as a negative regulator of cell proliferation, arresting the cell cycle at the G0/G1. Hypertension led to decreased activity of CaCC in the basilar smooth muscle cells of hypertensive rats, which was associated with upregulation of Ca^2+^/calmodulin-dependent protein kinase II (CaMKII) activity and downregulation of TMEM16A expression [29]. Thus, the importance of CaCCs in the maintenance of normal vascular tone needs further investigation, especially during aging. 

The contractile phenotype of smooth muscle myocytes is common in adult tissue. The hallmarks of the contractile phenotype are slow proliferation and a physiologically adequate response to vasoactive regulators such as acetylcholine and norepinephrine [32]. As mentioned before, age-dependent changes in vascular tone regulation are associated with a progressive decline in vascular responses to constrictors (for detailed information, see [2]). These results indicate that the decrease in CaCCs activity is at least partially involved in the reduced contraction of VSMC during aging (Figure 1B). 

As early as 40 years ago, it was observed that sympathetic nerves have trophic influence on arterial structure and function, and denervation increases non-specific sensitivity to vasocontracting agents independently of age [33]. CaCCs blockers suppressed methoxamine-induced arterial contraction and methoxamine-induced depolarization of VSMC to a greater degree in young (1- to 2-week-old) rats than in adult (2- to 3-month-old) rats [16]. Chronic sympathectomy diminished this age-dependent difference and resulted in a higher Cl^−^ contribution to arterial contraction in 2-month-old rats along with enhanced expression of TMEM16A protein [16].

Some studies evaluating protein expression of L-VDCC showed downregulation of L-VDCC protein expression [34,35], while others reported no change in protein expression [36,37]. Thus, various results were published about the protein expression of L-VDCC. To further evaluate the physiological mechanisms, the blood pressure response to the L-VDCC inhibitor was measured. In this study, the drop in blood pressure after nifedipine (L-VDCC inhibitor) application was similar in young SHR and WKY [5]. The drop in blood pressure after nifedipine was augmented in 42-weeks-old SHR but was unchanged in 42-weeks-old WKY [5], which would point to an accelerating influence of hypertension but not aging. Indeed, another group proved that L-VDCC current density were also significantly lower in old arteries (−2.77 ± 0.45 pA/pF) compared to young arteries (−4.5 ± 0.40 pA/pF) and the sensitivity of small mesenteric arteries to L-VDCC inhibitors was shifted to the right, meaning that the L-VDCC inhibitors were less effective in inhibiting the KCl-induced depolarization [38]. For more details about alterations of L-VDCC in aging, see [39]. 

A recent study by Jensen et al. [40] showed that the silencing of TMEM16A had no impact on the blood pressure and heart rate, but tail arteries from TMEM16A-silenced mice constricted less to contraction induced by adrenergic stimulation as well as to depolarization induced by elevated K^+^ [40]. Surprisingly, tail arteries with silenced TMEM16A protein expression were more sensitive to nifedipine-induced relaxation [40]. The down-regulation of TMEM16A in tail arteries was accompanied by the down-regulation of protein expression of vascular L-VDCC [40], pointing to the connection between L-VDCC and CaCCs as shown in Figure 1B. The arteries of adult animals have lower Ca^2+^ sensitivity during contractions than the arteries of young 1- and 2-week-old animals [41]. Denervation restored the Ca^2+^ sensitivity during agonist-induced contractions [41]. It is important to mention that denervated arteries contained less myosin light chain kinase and h-caldesmon and more extracellular-regulated kinases and p38 mitogen-activated protein kinases [41]. 

Taken together, current evidence about the CaCCs points towards age-dependent loss of CaCCs activity. It is plausible that this age-dependent decline of CaCCs activity is involved in the loss of contractility of VSMC during ontogenetic development and aging, and that during the contraction of VSMC, Ca^2+^ influx through various Ca^2+^ channels is connected with the activation of CaCCs and declines as well. The current knowledge presented above suggests that the Ca^2+^ influx and the activation of CaCCs decline with aging, as shown in Figure 1B.

## 3. Molecular Mechanisms of Endothelial Ca^2+^-Dependent Cl^−^ Channels

There are only a few recent studies that focus on the involvement of endothelial CaCCs in vascular tone regulation. The CaCCs represent an important pathway for the regulation of endothelium-derived factors. The resting membrane potential of human umbilical vein endothelial cells (HUVECs) was measured and reached −47 ± 2 mV [42]. Unfortunately, to our knowledge, there is no information about the equilibrium potential for Cl^−^ of endothelial cells. The [Cl^−^]i of endothelial cells was evaluated in HUVECs, where the [Cl^−^]i was 33.3 ± 2.6 mmol/L [43], which is slightly lower than the [Cl^−^]i of VSMC. 

Since the [Cl^−^]i of endothelial cells is slightly lower than that of VSMC, and the equilibrium potential for Cl^−^ of endothelial cells is unknown, the activation of CaCCs leaves open two possibilities, i.e., that CaCCs may promote depolarization and nitric oxide (NO) release from intact endothelium, or that CaCCs may cause hyperpolarization (Figure 1). NO is produced by NOS from L-arginine, and various mechanisms deploy their beneficial effects through the activation of endothelial NOS [44,45,46,47] or the inhibition of inducible NOS [48]. TMEM16A on endothelial cells are localized not only at the plasma membrane but also at the mitochondrial membrane; thus, it is important to distinguish which channels are influenced while carrying out a study with CaCCs.

The [Cl^−^]i in the VSMC was reported to be about 35 mM [21]. If the [Cl^−^]i of endothelial cells of mesenteric arteries is similar to that in HUVECs, where the [Cl^−^]i was found to be 33.3 ± 2.6 mmol/L [43], the equilibrium potential for Cl^−^ would be less negative than the resting membrane potential. Under such conditions, an activation of Cl^−^ channels will depolarize the membrane [49], similar as is proposed for VSMC. Acetylcholine-induced hyperpolarization observed in VSMC is due to conduction through the myoendothelial gap junctions [50], the endothelial Ca^2+^-activated K^+^ channels of intermediate conductance (IK_Ca_) and small conductance (SK_Ca_) currents promote the endothelium-derived hyperpolarization phenomenon, and the Ca^2+^-dependent Cl^−^ current would restrain it [49]. Indeed, the study of Skofic Mauer et al. [51] carried out on TMEM16A localized at the plasma membrane of pulmonary arterial endothelial cells has shown that increased TMEM16A activity interferes with background endothelial NOS activity [51]. This leads to instable NO production [51]. Stable NO production is essential for maintaining low vascular tone.

If the activation of TMEM16A should create hyperpolarization, the equilibrium potential for Cl^−^ needs to be negative compared to physiological voltage to elicit Cl^−^ influx. The intracellular Cl^−^ concentration in endothelial cells of pressurized mesenteric arteries can only be estimated because there is no information in the literature yet. When considering the arterial membrane potential of mesenteric arteries pressured to 80 mmHg and with 122 mM Cl^−^ in the bath solution, the membrane potential was ~−32.2 mV [31]. Under these conditions, the [Cl^−^]i has to be lower than 34.8 mM for CaCCs currents to produce hyperpolarization in endothelial cells [31].

There are also results for reduced CaCCs activity. These results imply that reduced TMEM16A channel activity causes membrane hyperpolarization and subsequent [Ca^2+^]i increases in brain capillary endothelial cells, and the activity of TMEM16A CaCCs is involved in the regulation of the trans-endothelial permeability in the blood–brain barrier [14]. Although the [Cl^−^]i concentration in brain capillary endothelial cells is not known, it is estimated to be 30–40 mM in vascular endothelial cells [20]. Membrane hyperpolarization after CaCCs inhibition also facilitated Ca^2+^ influx through non-selective cation channels regulating brain capillary endothelial cells’ proliferation and migration [14]. The TMEM16A knockdown attenuated brain capillary endothelial cells proliferation and migration as well [14]. TMEM16A is upregulated after ischemic stroke, and knockdown of TMEM16A in brain endothelial cells leads to attenuation of inflammation through the decrease in activation of nuclear factor kappa-light-chain-enhancer of activated B cells (NF-κB) and intercellular adhesion molecule 1 (ICAM-1) [52]. NF-κB and ICAM-1 are essential to elicit ischemia-induced blood–brain barrier damage [52]; thus, the CaCCs inhibition could have a beneficial effect in the treatment of ischemic stroke.

When the CaCCs were activated by acetylcholine and ATP, they elicited vasorelaxation by increasing the [Ca^2+^]i concentration in endothelial cell [31]. The CaCCs activation increases the activity of the transient receptor potential cation channel subfamily V member 4 (TRPV4) channels, which induces an increased driving force for Ca^2+^ entry into the endothelial cell [31]. In a mouse model of endothelial cell-specific TMEM16A knockout, the activation of TMEM16A channels was disrupted, and their outward rectification was reduced, leading to impaired vasorelaxation and increased blood pressure [31].

As mentioned above, the [Cl^−^]i has to be lower than 34.8 mM for CaCCs currents to produce hyperpolarization in the endothelial cells of mesenteric arteries [31]. Interestingly, inflammatory factors like tumor necrosis factor-α (TNF- α)caused a decrease in the [Cl^−^]i to 23.1 ± 1.9 mmol/L in HUVECs [43]. Furthermore, in patients with hypercholesterolemia, there was a decrease in the [Cl^−^]i monocytes/macrophages, leading to enhanced pro-inflammatory cytokine production and the acceleration of foam cell formation, leading to progression of atherosclerosis [53]. These results concerning the decrease in the [Cl^−^]i in endothelial cells that promotes inflammation and foam cell formation could partially explain the higher risk of mortality under a low Cl^−^ environment [54]. The low serum Cl^−^ level is an independent predictor of mortality in hypertensive patients or in patients with heart failure [54]. 

It is important to mention that the TMEM16A protein that forms CaCCs is also localized on the mitochondria of pulmonary endothelial cells. Activation of TMEM16A induces apoptosis by activating the mitochondrial reactive oxygen species (ROS)–p38–caspase-3 pathway [55]. CaCCs activation through increased TMEM16A protein levels in pulmonary arterial hypertension (PAH) promotes apoptosis in endothelial cells from PAH patients compared with control subjects [55]. Apoptosis in endothelial cells exposed to hypoxia is enhanced, but this augmentation cannot be attributed to enhanced CaCCs activity or protein levels associated with increased expression because neither siRNA nor a CaCCs inhibitor (4,4′-Diisothiocyano-2,2′-stilbenedisulfonic acid, DIDS) had any effect on the untreated hypoxic endothelial cells [55].

In endothelial cells, inflammation causes a decrease in the [Cl^−^]i [43], which might change the measured and estimated outward Cl^−^ current [31] to an inward Cl^−^ current through CaCCs. To our knowledge, there are currently no data available concerning the changes in the [Cl^−^]i in endothelial cells during ontogenetic development. Chronic low-grade inflammatory processes are part of aging and may be defined as “inflammaging” [56]. Aging increases the caloric intake, which, in turn, activates and supports chronic low-grade inflammation [56].

These age-dependent changes of inflammatory processes could help to explain discrepancies in the results of various studies. Despite the fact that our knowledge of the important role of chloride channels in the regulation of homeostasis and physiological processes is already 100 years old, a pharmacological treatment of cardiovascular diseases that would involve alterations in the chloride channels remains elusive.

## 4. Relation of Sympathetic Nervous Activity, Ca^2+^-Dependent Cl^−^ Channels, and Aging

Very recent findings point to an interesting relationship between SNA, NOS activity, and CaCCs during ontogenetic development. Aging impairs the NOS activity [3]. A recent experimental study revealed that in SHRs, aging leads to deterioration of the NOS system; conversely, the NOS system in WKY rats maintains its endothelial functions, leading to maintenance of optimal blood pressure levels even in later periods of life [4]. It is hypothesized that increased NOS activity in rostral ventrolateral medulla (RVLM) causes inhibition of renal sympathetic nerve activity (RSNA), which further reduces NOS activity in the kidney [57]. Indeed, NO formed in neurons in the RVLM inhibits the central sympathetic nerve activity innervating the kidney, leading to renal vasodilatation and increased renal blood flow [58]. The renal vasodilation might be balanced by reduction in neuronal and/or endothelial NOS activity [57]. Conversely, when the NOS activity is decreased in the RVLM, the RSNA is enhanced together with augmented renal neuronal and/or endothelial NOS activity, suggesting that NOS activity and SNA display organ-specific alterations [57]. Indeed, it was shown that NO has a biphasic effect on cardiovascular activity in RVLM [59]. High levels of NO in RVLM decrease arterial pressure, whereas low levels of NO increase arterial pressure [59]. Furthermore, an age-dependent decrease in the activity of NOS in the vasculature and nervous system leads to elevation of RSNA [7]. The enhanced RSNA activity may decrease the expression of CaCCs during ontogenetic development since, as already mentioned, the sympathetic nervous system has trophic influence on CaCCs in the post-natal period, leading to a post-natal decline in the contribution of Cl^−^ channels to arterial contraction [16]. In the mature arterial wall, the contribution of Cl^−^ to arterial contraction is smaller [16]. Experiments on aged animals should be carried out to disclose mechanisms involved in the regulation of CaCCs related to SNA and NOS. These findings require further investigation to reveal more details about this proposed pathway to prove the connection. 

## 5. Scramblase Activity of Ca^2+^-Dependent Cl^−^ Channels and Endothelium-Dependent Contraction

Prostaglandins, as cyclooxygenase (COX) products, are important regulators of vascular function, growth, platelets aggregation, etc. The substrate for COX is the arachidonic acid which is being formed from membrane phospholipids by phospholipase A_2_ (PLA_2_). From arachidonic acid, COX produce prostaglandins, leukotrienes, and even factors that evoke endothelium-dependent contraction (EDC). COX-produced prostaglandins, such as thromboxane A_2_ and prostaglandins H_2_, E_2_, and F_2α_, represent one group that is causing EDC.

The effect of prostaglandin-related EDC in the regulation of vascular tone increases with aging [12]. Hypertension accelerates EDC utilization in the regulation of vascular tone at a younger age, as was shown in the norepinephrine-induced contraction of the isolated rat femoral artery [12]. EDC is also important in humans because indomethacin (COX inhibitor) completely reversed the blunted vasodilation to acetylcholine by restoring NO availability [60]. 

Isoprostanes are prostaglandins-like compounds produced in vivo through the peroxidation of fatty acids and include H_2_-, F_2_-, E_2_-, D_2_-, A_2_-, J_2_-isoprostanes, isothromboxanes, and isoketals [61]. The serum/plasma fatty acid profile has a high predictor value of an individual’s health. The precise composition of phospholipids available to PLA_2_ and subsequent COX activity is modulated by various factors, including phospholipid scramblase activity. Activation of scramblases results in the fast and passive transbilayer movement of lipids and leads to the externalization of negatively charged phosphatidylserine molecules to the outer leaflet of membrane, which is an important step in extracellular signalling [62]. 

Phospholipid scramblase renders the cellular membranes more susceptible to PLA_2_ signalling [63]. Activation of PLA_2_ and lipid peroxidation enhance ion currents via TMEM16F and lead to increased TMEM16F phospholipid scramblase activity in platelets [15,64]. In general, it is assumed that all members of the TMEM membrane protein family maintain a certain level of scramblase activity as well as CaCCs activity.

Falzone et al. [65] propose that lipid scrambling is primarily determined by the ability of TMEM16 proteins to thin the membrane so that lipids mainly interact with the surface without penetrating deep within its narrow and hydrophilic interior. The TMEM16 proteins scramblase activity is modulated by two signals: first is the binding of Ca^2+^ that facilitates the opening of the groove; and the second involves the properties of the cell membrane, which determine whether the scramblase is able to thin the cell membrane enough to enable lipid flipping [65].

The phospholipid scramblase activity of CaCCs leads to the activation of coagulation processes since the TMEM16F protein was required for phosphatidylethanolamines and phosphatidylserines externalization during thrombin activation [66]. In Scott syndrome, the TMEM16F protein is the mutated protein behind the defected phospholipid scrambling activity, leading to impaired exposure of phosphatidylserine on the platelet membrane.

The function of CaCCs in angiogenesis has not yet been evaluated in many studies. A recent study found that after the expression of TMEM16F was decreased in TMEM16F-deficient mice, the development of retinal angiogenesis was impaired [67]. The decrease in angiogenesis in TMEM16F-deficient mice was evidenced by fewer vascular loops and larger loop areas [67]. 

The scrambling activity of CaCCs is important for angiogenesis and coagulation and could have an impact on the regulation of vascular tone through the modulation of PLA_2_ activity and COX activity.

## 6. Ca^2+^-Dependent Cl^−^ Channels Involved in Various Diseases

The lungs of patients with COVID-19 contain syncytia that result from the activation of the severe acute respiratory syndrome coronavirus 2 (SARS-CoV-2) spike protein at the cell plasma membrane [68]. The activity of TMEM16F protected against virus replication and associated cytopathicity [68]. SARS-CoV-2 induced phosphatidylserine scrambling, as was shown in experiments with the application of the SARS-CoV-2 spike pseudotyped virus [69]. SARS-CoV-2 evokes a cytosolic Ca^2+^ elevation and TMEM16F-dependent phosphatidylserine externalization [69], making a connection between SARS-CoV-2 and CaCCs. 

Studies on the TMEM16 protein family were performed before the TMEM16A was identified as a CaCC channel, and these studies highlighted the function of CaCCs in tumorigenesis. In esophageal squamous cell cancer, gastrointestinal stromal tumor, head and neck squamous cell carcinoma, and pancreatic and breast cancers, it has been suggested that the amplification of TMEM16A could serve as a biomarker [70]. TMEM16A is a part of human chromosome 11q13 amplicon and is the most frequently amplified chromosomal regions in human cancers [70]. The over-expression of TMEM16A correlates with shorter overall survival rate and poor prognosis [70,71]. Interestingly, TMEM16A is not a universal marker of poor prognosis. In some subtypes of cancers, such as human-papilloma-virus-positive head and neck squamous cell carcinoma, pancreatic neuro-endocrine tumors, and estrogen-receptor-positive and human epidermal growth factor receptor 2-positive breast cancers, there is no over-expression of TMEM16A [71]. 

High levels of TMEM16A correlate with the size of the tumor and the presence of distant metastasis [71]. In non-small-cell lung cancer, TMEM16A expression at the RNA and protein level was significantly increased in tumor tissues compared to the paraneoplastic tissues [72]. Enhanced expression of TMEM16A correlated with the increased recurrence rate [72]. Inhibition of TMEM16A leads to improved clinical outcomes by chemotherapy in breast cancer [73]. The over-expression of TMEM16A was linked to the clinical stage of cancer in human prostate carcinoma and gastrointestinal stromal tumors [71]. The inhibition of TMEM16A represent a new possible therapeutic target in the treatment of various types of cancer [73] because TMEM16A contributes to “sustained proliferation”, which is a hallmark of cancerous tissue [71]. 

As was shown that in cell proliferation and apoptosis, the levels of Cl^−^ are playing an important role [70,71,72,73,74]. The investigation of the influence of Cl^−^ in the cardiovascular system has been going on for more than one century, but there are still no known therapeutic targets involving Cl^−^ channels or transporters for cancer treatment or the treatment of cardiovascular diseases [74]. 

## 7. Conclusions

Vascular Cl^−^ signaling, in general, has not yet received adequate attention. It has been established that the opening of the CaCCs lead to efflux of Cl^−^, triggering the depolarization of VSMCs. Few results suggest that aging causes the downregulation of CaCCs and/or decline in CaCCs activity. The decline of CaCCs distribution in vasculature already starts postnatally due to the trophic effect of sympathetic nerves, but further studies are needed to disclose whether the decline in CaCCs distribution is preserved during whole ontogenetic development and aging.

Very few studies have focused on the function of CaCCs in endothelial cells. Depending on the endothelial [Cl^−^]i, the activation of CaCCs on endothelial cells causes either depolarization or hyperpolarization of the endothelial cells and thus influences the release of endothelium-dependent relaxing and contracting factors. Furthermore, expected outward rectification of CaCCs might be changed under inflammation because the production of inflammatory autacoids (such as TNF-α) causes a decrease in the endothelial [Cl^−^]i. TMEM16 protein family members that provide the molecular basis of CaCCs were proven to be expressed on the plasma membrane of VSMC and endothelial cells, as well as on the mitochondrial membrane. 

The activation of CaCCs on the plasma membrane serves as phospholipid scramblase, leading to the externalization of negatively charged phosphatidylserine molecules to the outer leaflet of membrane.

It is tempting to imagine that the monitoring of CaCCs activity and the alteration of the [Cl^−^]i could predict a shift from a physiological to a pathophysiological state of VSMCs and endothelial cells, and thus that CaCCs activity could serve as a marker for pathological changes in the vasculature. However, the major conclusion is that the function of CaCCs in ontogenetic development and aging is not well understood and remains controversial. Some mechanisms discussed in this review are hypothetical and were extrapolated from only a few studies. Unfortunately, there are no more studies (and not a single review) that focus on CaCCs and aging. Thus, the function of CaCCs in aging requires further extensive and rigorous investigation.
